# CLAIMS-BASED FRAILTY INDEX FOR IDENTIFYING MODERATE-TO-SEVERE DEMENTIA IN MEDICARE BENEFICIARIES

**DOI:** 10.1093/geroni/igae098.1262

**Published:** 2024-12-31

**Authors:** Chan Mi Park, Stephanie Denise Sison, Ellen McCarthy, Sandra Shi, Dae Hyun Kim

**Affiliations:** Hebrew SeniorLife, Boston, Massachusetts, United States; University of Massachusetts Chan Medical School, Worcester, Massachusetts, United States; Harvard Medical School, Boston, Massachusetts, United States; Harvard Medical School/Hebrew SeniorLife, Boston, Massachusetts, United States; Hebrew SeniorLife, Boston, Massachusetts, United States

## Abstract

Identifying dementia severity from administrative claims poses challenges. This abstract discusses developing and validating a claims-based frailty index (CFI) cut-point for identifying moderate-to-severe dementia among Medicare beneficiaries, using data from the National Health and Aging Trends Study (NHATS) and Medicare Current Beneficiary Survey (MCBS). We used 2015 data from NHATS linked to Medicare claims to calculate CFI and determine the optimal cut-point that maximizes sensitivity and specificity in identifying moderate-to-severe dementia based on the Functional Assessment Staging. We validated this measure on an independent sample of community-dwelling Medicare beneficiaries with dementia from MCBS. Performance metrics included sensitivity, specificity, and positive and negative predictive values. From the NHATS development cohort (n=814), the CFI cut-point of 0.280 demonstrated strong performance (C-statistic of 0.78) in differentiating moderate-to-severe dementia cases, achieving the maximum sensitivity of 76.9% and specificity of 62.8%. This cut-point correlated significantly with dementia indicators, including higher rates of functional disability, dementia medication use, mortality, and nursing home admissions. Subsequent validation using the MCBS cohort (n=658) supported the cut-point’s utility in identifying moderate-to-severe dementia, with modest sensitivity of 0.49 and high specificity of 0.80. These two pivotal studies suggest that the CFI, with a cut-point of 0.28, is a robust tool for identifying moderate-to-severe dementia in Medicare claims data. By offering an evidence-based metric, this research facilitates a more targeted approach to allocating healthcare resources and planning care interventions for dementia patients, bridging a critical gap in the absence of direct clinical measures of dementia severity.

